# Characterization of *LhSorP5CS*, a gene catalyzing proline synthesis in Oriental hybrid lily Sorbonne: molecular modelling and expression analysis

**DOI:** 10.1186/s40529-017-0163-0

**Published:** 2017-01-18

**Authors:** Le Wang, Zhihong Guo, Yubao Zhang, Yajun Wang, Guo Yang, Liu Yang, Ruoyu Wang, Zhongkui Xie

**Affiliations:** 1 Gaolan Station of Agricultural and Ecological Experiment, Northwest Institute of Eco-Environment and Resources, Chinese Academy of Sciences, Lanzhou, 730000 China; 2 University of Chinese Academy of Sciences, Beijing, 100049 China

**Keywords:** *LhSorP5CS* gene, Homology modeling, Proline, Abiotic stress, *Lilium* spp.

## Abstract

**Background:**

Abiotic stresses negatively affect plant growth and flower production. In plants, P5CS proteins are key enzymes that catalyzed the rate-limiting steps of proline synthesis, and proline is a well-known osmoprotectant that is closely related to abiotic stress tolerance. However, information about the P5CS genes, their effects on proline accumulation, and their role in abiotic stress tolerance in *Lilium* is still lacking.

**Results:**

We isolated and characterized a novel gene (*LhSorP5CS*) from Oriental hybrid lily cultivar Sorbonne. Phylogenetic analysis indicated that *LhSorP5CS* is a member of the *P5CS* family. The three-dimensional structure of LhSorP5CS predicted by homology modeling showed high similarity to its correspondant human P5CS template. Further gene expression analysis revealed that *LhSorP5CS* expression was up-regulated by NaCl, mannitol, and ABA, and that stress-exposed plants accumulated proline at a significantly higher level than in the control.

**Conclusions:**

*LhSorP5CS* characterized in this study is involved in proline synthesis in lily, and that it might play an important role in abiotic stress tolerance. However, there should be other *P5CS* homologues in the lily genome, and some of them could be highly stress-induced and more important for proline accumulation. Future studies on *P5CS* family genes would be of great importance to proline-related stress tolerance in lily.

**Electronic supplementary material:**

The online version of this article (doi:10.1186/s40529-017-0163-0) contains supplementary material, which is available to authorized users.

## Background

Abiotic stresses such as salinity, drought, heat, and water-logging negatively affect plant growth. These environmental constraints further restrict the range of sites that are suitable for cultivation, and cause decreased agricultural productivity around the world (Zhu 2001). To protect themselves against adverse conditions, plants have evolved many physiological, cellular, and molecular mechanisms (Tan et al. 2013). One of the most well-studied protective mechanisms relates to proline metabolism. Stress responses in plants are often accompanied by the accumulation of proline in different tissues (Verbruggen and Hermans 2008). The accumulated proline can function as an intracellular osmolyte (Chinnusamy et al. 2005), a scavenger for reactive oxygen species (ROS) (Matysik et al. 2002), a maintainer of cell structure (Verslues et al. 2006), and a signaling molecule that primes multiple stress response pathways (Maggio et al. 2002). More recent findings have connected proline to redox status (Sharma et al. 2011; Shinde et al. 2016), and proline was considered as a storage of both energy and reducing potential (Szabados and Savoure 2010). Besides, proline metabolism was also involved in programmed cell death and plant-pathogen interaction. Accumulation of proline was observed at the site of infection during incompatible interaction in Arabidopsis (Fabro et al. 2004).

The metabolic pathways for proline synthesis have been deciphered and proven to be evolutionarily conserved from bacteria to higher organisms (Rai and Penna 2013). In plants, two routes for proline synthesis are known to exist, a glutamate pathway and an ornithine pathway (Adams and Frank 1980; Delauney and Verma 1993). However, the glutamate-derived pathway is the major route for proline synthesis under stress conditions (Trovato et al. 2008). In this pathway, glutamate is reduced by Δ1-pyrroline-5-carboxylate synthetase (P5CS) to form γ-glutamate-semialdehyde (GSA) in a manner that depends on ATP and NADPH, then GSA cyclized itself to form P5C (Liang et al. 2013). These reactions are rate-limiting steps in proline synthesis and are catalyzed by P5CS; thus, *P5CS* genes play an important role in stress tolerance.

Given that P5CS plays such an important role in proline synthesis, many attempts to improve stress tolerance by increasing proline production have centered on *P5CS* genes. For example, overexpression of *P5CSF129A* resulted in increased proline production and enhanced salt tolerance in *indica* rice (*Oryza sativa* L.) (Kumar et al. 2010). In citrus species, plants overproduced proline exhibited superior osmotic adjustment and higher photosynthetic rates under drought stress (Molinari et al. 2004).

In the present study, we cloned the full-length cDNA sequence of a *P5CS* gene (*LhSorP5CS*) from the Oriental hybrid lily cv. Sorbonne (*Lilium* spp.) by rapid amplification of cDNA ends (RACE). Phylogenetic analysis of the deduced LhSorP5CS amino acid sequence by comparison with other P5CS proteins in plants indicated that LhSorP5CS was a member of the P5CS family. We investigated the expression of *LhSorP5CS* in different tissues and in response to salt, drought, and abscisic acid (ABA) stresses by means of real-time quantitative polymerase chain reaction (qPCR). To our knowledge, *LhSorP5CS* is the first *P5CS* gene that has been functionally characterized in *Lilium*.

## Methods

### Plant growth conditions

The Oriental hybrid lily cultivar Sorbonne was planted in the greenhouse of the Gaolan Agricultural and Ecological Experiment Station, in northern China. Bulbs 10–12 cm in diameter were grown in plastic pots containing peat and kept under a 16 h light/8 h dark photoperiod at 22 °C.

### Total RNA extraction and first-strand cDNA synthesis

Total RNA was extracted with RNAprep Pure Plant kits (TIANGEN Corporation, Beijing, China) following the manufacturer’s instructions. We used 1 µg of total RNA for first-strand cDNA synthesis. First-strand cDNA was synthesized using the PrimeScript first-strand cDNA synthesis kit (Takara, Dalian, China) or the SMARTer RACE cDNA Amplification Kit (for RACE cloning; Clontech, USA). Synthesized cDNA was subsequently diluted to a final concentration of 20 ng µL^−1^ with nuclease-free water for use in cloning.

### Full length cloning of *LhSorP5CS*

We designed a degenerate primer set (P5CS-Deg-F: 5′-GGNATHTTYTGGGAYAAYGA-3′; P5CS-Deg-R: 5′-GTYTCCATNGCRTTRCANGC-3′) based on conserved regions for plant P5CS enzymes. PCR was performed in a 25-µL reaction mixture containing 20 ng of template cDNA, 200 µmol L^−1^ of dNTPs, 1.0 µmol L^−1^ of each primer, and 1.25 U of TransTaq HiFi DNA polymerase (TransGen, Beijing, China). PCR products were gel-purified, ligated into the pMD-18T vector (Takara, Dalian, China), and sequenced (Sangon, Shanghai, China). Based on the partial coding sequence that we obtained, we designed gene-specific primers for 5′-RACE (5RACE-GSP-1: 5′-GGTCGGCCTTCAACTCCATCGCC-3′; 5RACE-GSP-2: 5′-AGCCCCGCTAAGCTGTCATTGTCCC-3′) and 3′-RACE (3RACE-GSP-1: 5′-GATCTCGTGATTCCGAGGGGTAGC-3′; 3RACE-GSP-2: 5′-CGCCAAGACAGATTACCCAGCAGC-3′). The full-length sequence was obtained by assembling the 5′-RACE sequence, the partial coding sequence, and the 3′-RACE sequence.

### In silico analysis of *LhSorP5CS*

The open reading frame (ORF) of the full-length cDNA of the *LhSorP5CS* sequence was predicted using the ORF finder. Homology of the deduced P5CS protein with other members of this family was verified by querying the NCBI database using the BLAST software. Multiple alignment of sequences was performed using the Clustal W program. A phylogenetic tree of the P5CS proteins was constructed using the Mega 4.1 software (Tamura et al. 2007) using the neighbor-joining method.

### Three-dimensional structure prediction by homology modeling

Prediction of 3D structure was carried out via accessing Swiss-Model(Schwede et al. 2003), a web-based protein structure prediction tool. Superposition of the built LhSorP5CS model on the template was achieved by Swiss PDB viewer programme. The root mean square deviation (RMSD),which measure the average distance between the backbone atoms of superposed proteins, was calculated using the PDBeFold programme (Krissinel 2007). Evaluation of stereochemical quality of the LhSorP5CS model was carried out by using Procheck V.3.5.4 (Laskowski et al. 1993).

### Expression analysis of *LhSorP5CS* in different tissues and under various stress treatments

Tissues (root, stem, leaf, petal, and scale) were sampled from 2-month-old fully bloomed plants and used as the materials for tissue-specific expression analysis. For exogenous stress treatments, the bulbs of 30-day-old seedlings were drenched with 500 mL solutions containing 500 mM mannitol, 500 mM NaCl, or 2 µM ABA. Leaf samples were taken 0, 2, 4, 8, 12, and 24 h after the stress treatments. All samples were frozen immediately in liquid nitrogen and kept in a refrigerator at −80 °C until use.

After RNA extraction, first strand cDNA synthesis of templates for qPCR was carried out using the HiScript II Q RT SuperMix for qPCR kits (Vazyme Biotech, Nanjing, China), and contamination with genomic DNA was removed by using the gDNA wiper following the manufacturer’s protocol. Real-time qPCR was performed using an MX3000P qPCR thermocycler (Stratagene, USA). The Oriental hybrid lily cv. Sorbonne *polyubiquitin4* gene (GenBank accession no. DW718023) was used as the reference gene (Yamagishi 2011). The P5CS-specific primer set (P5CS-q-F: 5′-GCTTTCATCAGGGACGCCAAG-3′; P5CS-q-R: 5′-CAACACCAACCGCACCAGAAG-3′) was designed using OligoArchitect (Fourie et al. 2014), online software developed by Sigma-Aldrich. qPCR was performed using the AceQ qPCR SYBR Green Master Mix kit (Vazyme Biotech, Nanjing, China) according to the manufacturer’s instructions. The following protocol was used for amplification: pre-denaturation for 10 min at 95 °C, followed by 40 cycles of 94 °C for 15 s and 60 °C for 30 s. Relative changes in gene expression were analyzed by using the 2^−ΔΔCT^ method, and the C_T_ values used were from three independent biological replicates.

### Proline content determination

We determined the free proline content according to the method proposed by Bates et al. (1973). In summary, 0.5 g fresh weight (FW) of leaf tissue was homogenized in 3% sulfosalicylic acid solution using a mortar and pestle. The homogenate was then filtered through Whatman 42 filter paper. We then added 2 mL of 2.5% acidic ninhydrin and 2 mL of acetic acid to 2 mL of the filtrate. After boiling for 1 h, the mixture was placed on ice and proline was extracted using 4 mL of toluene. The proline content per unit FW was calculated based on the absorbance readings at 520 nm using a UV-1200 spectrometer (Macy, Shanghai, China). Pure proline was used as the analytical standard.

### Statistical analysis

All experiments were repeated three times, with three replicates each time. We used version 8 of the SAS software for statistical analysis and the Origin Pro 8.1 software to plot the data.

## Results

### Molecular cloning and sequence analysis of *LhSorP5CS*

The cloned full-length *LhSorP5CS* cDNA contained 2645 bp, comprising a 5′ untranslated region (UTR) of 170 bp, a predicted ORF of 2139 bp, and a 3′ UTR of 196 bp. The ORF encoded a protein of 712 amino acids with a theoretical molecular mass of 77.3 kDa and a predicted isoelectric point (pI) of 5.71. The *LhSorP5CS* cDNA sequence has been submitted to Genbank and deposited with the accession number KU057356.

Multiple alignment showed that the deduced LhSorP5CS amino acid sequence exhibited high homology with other P5CS proteins from plants, such as MaAAAP5CS (83%), TaP5CS (77%), OsP5CS1 (79%), and ZmP5CS (79%). The LhSorP5CS protein sequence was compared with homologues from *Oryza sativa* and *Arabidopsis thaliana* (Additional file 1: Figure S1), and conserved regions included the ATP binding site, two Leu-rich domains, the Glu-5-kinase domain, the NAD(P)H binding domain, and the GSA-DH domain. A conserved phenylalanine residue (Phe, at position 125), which functioned in proline feedback inhibition, was also found in LhSorP5CS.

**Fig. 1:**
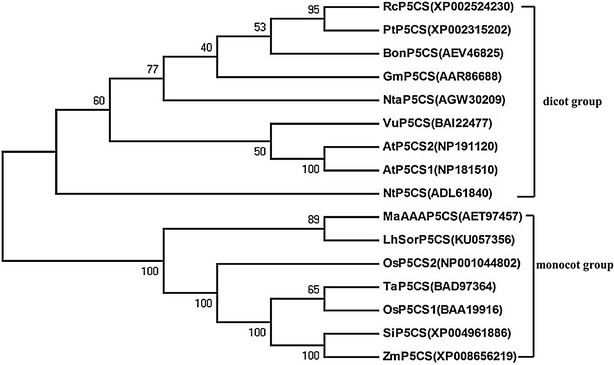
Phylogram of LhSorP5CS and other P5CSs from different species. Phylogenetic relationship of LhSorP5CS with AtP5CS1 (*Arabidopsis thaliana* P5CS1), AtP5CS2 (*A. thaliana* P5CS2), OsP5CS1 (*Oryza sativa* P5CS1), OsP5CS2 (*O. sativa* P5CS2), RcP5CS (*Ricinus communis* P5CS), PtP5CS (*Populus trichocarpa* P5CS), BonP5CS (*Boehmeria nivea* P5CS), GmP5CS (*Glycine max* P5CS), NtaP5CS (*Nitraria tangutorum* P5CS), VuP5CS (*Vigna unguiculata* P5CS), NtP5CS (*Nicotiana tabacum* P5CS), MaAAAP5CS (*Musa acuminata* AAA Group P5CS), TaP5CS (*Triticum aestivum* P5CS), SiP5CS (*Setaria italica* P5CS), and ZmP5CS (*Zea mays* P5CS). *Numbers in brackets* refer to the sequences accession number in GenBank

A phylogenetic tree was built to analyze the evolutionary relationship of the LhSorP5CS protein with P5CS proteins from other species. Phylogenetic analysis revealed that the P5CS proteins in plants clustered into two major groups: the monocot group and the dicot group. LhSorP5CS was most closely related to MaAAAP5CS in the monocot group (Fig. 1). These findings suggest that the *LhSorP5CS* that we cloned is a new gene in the *P5CS* family, and that the putative LhSorP5CS might be involved in proline synthesis in lily.

### Predicted three-dimensional structure of the LhSorP5CS protein

**Fig. 2:**
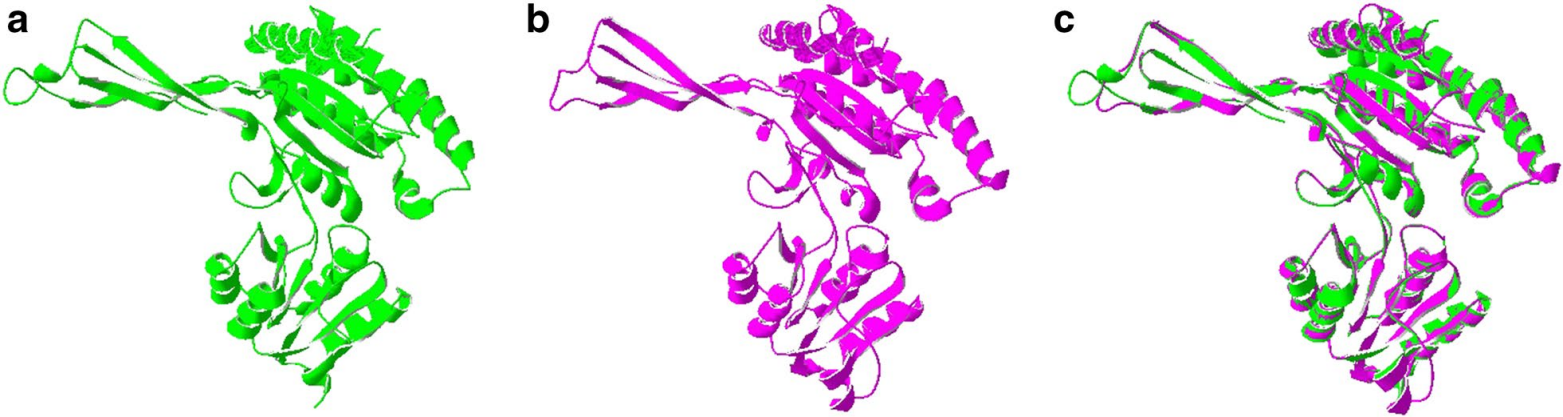
Three dimensional structure of LhSorP5CS generated by homology modeling with the human P5CS as template. **a** Structure of the human P5CS used as template. **b** Predicted structure of LhSorP5CS. **c** Superposition of LhSorP5CS and the human P5CS protein shows highly similar structure between the two homologs

The deduced LhSorP5CS protein sequence was submitted to server to BLAST against the Swiss-Model template library to obtain the best template. The searching result revealed that human P5CS (2h5g.1.A) which share 50.12% identity with LhSorP5CS was the best alignment. Thus, the human P5CS protein was used as template (Fig. 2a) to build the LhSorP5CS 3D model (Fig. 2b). Superposition (Fig. 2c) of the built LhSorP5CS model with the human P5CS template showed that 3D structure of these two proteins were highly similar, and the calculated RMSD of the superposed proteins was 0.45. Ramachandran plot (Additional file 2: Figure S2) showed that 90.3% of residues in the LhSorP5CS model were in the most favored regions, 8.7% in the additional allowed regions, 0. 5% in the generously allowed regions; while only 0.5% of residues were in the disallowed regions. The result that over 90% of the residues were in the most favored regions indicated that the LhSorPR5 model presented was well built.

### Expression analysis

#### Tissue-specific expression of *LhSorP5CS*

**Fig. 3:**
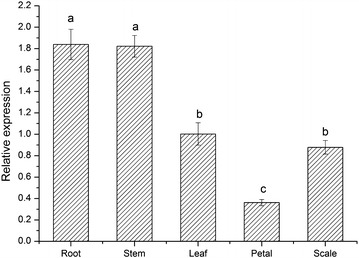
Tissue-specific expression of *LhSorP5CS* from Oriental hybrid lily cv. Sorbonne (*Lilium* spp.). Transcript levels of the target gene were normalized against the *polyubiquitin4* internal control. Values are mean ± standard deviation (SD) for three replicates. *Bars* labeled with *different letters* differ significantly (*P* < 0.05, *t* test)

Figure 3 shows that *LhSorP5CS* expression was detected in all five tissues (root, stem, leaf, petal, and scale), but the abundance of transcripts was significantly higher in the root and stem tissues than in the other tissues. The levels of *LhSorP5CS* transcripts in root and stem tissues were 5.08 and 5.03 times, respectively, that in the petal tissue, which had the lowest expression. The abundance of *LhSorP5CS* transcripts in petal tissue was also significantly lower than that in leaf and scale tissues (which were 2.77 and 2.41 times the petal level, respectively).

### Response of *LhSorP5CS* expression to abiotic stresses

**Fig. 4:**
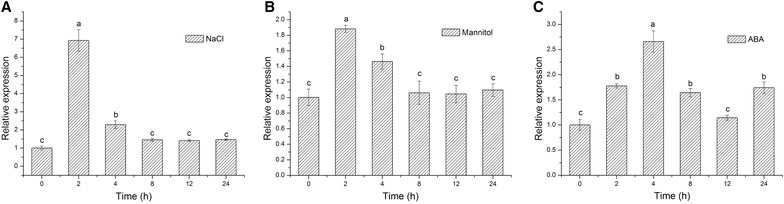
*LhSorP5CS* expression pattern in response to abiotic stresses. *LhSorP5CS* transcript level was quantified in leaves of the Oriental hybrid lily cv. Sorbonne (*Lilium* spp.) under the **a** NaCl, **b** mannitol, and **c** ABA treatments. Values were determined by real-time qPCR, and normalized using transcript levels of *LhSorP5CS* in the leaves of unstressed plants (the control), which were set to a value of 1. Values represent the mean ± standard deviation (SD) for three replicates. *Bars* labeled with *different letters* differ significantly (*P* < 0.05, *t* test). Note that the y-axis scale differs greatly between graphs

We used the levels of *LhSorP5CS* transcripts to investigate whether *LhSorP5CS* expression was induced by salt, drought, or ABA stress. Figure 4a shows that *LhSorP5CS* expression was strongly and significantly induced by NaCl 2 h after treatment, reaching a level 6.92 times that in the control. Similarly, significant up-regulation of *LhSorP5CS* also occurred 2 h after mannitol treatment, to 1.88 times the control level (Fig. 4b); that is, the response was weaker than that to the NaCl treatment. Under ABA treatment, *LhSorP5CS* expression was significantly up-regulated by 2 h after treatment, reaching 1.77 times the control level, and then increased to 2.66 times the control level by 4 h after treatment (Fig. 4c); this was also weaker than the NaCl response. The rapid induction of *LhSorP5CS* in the presence of salt, drought, and ABA stress suggests that *LhSorP5CS* is an abiotic stress-response gene in lily.

### Proline accumulation

**Fig. 5:**
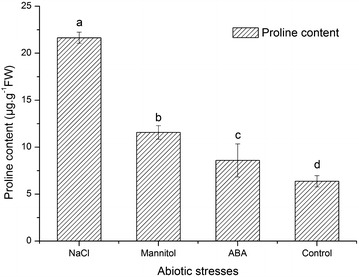
Proline contents in leaves of Oriental hybrid lily cv. Sorbonne (*Lilium* spp.) sampled 12 h after treating lily plants with different abiotic stresses. Values represent mean ± standard deviation (SD) for three replicates. *Bars* labeled with *different letters* differ significantly (*P* < 0.05, *t* test)

We also measured proline accumulation in leaves 12 h after the abiotic stress treatments, using the same samples used for qPCR. The NaCl, mannitol, and ABA treatments all significantly increased proline accumulation in the leaves compared with the control, with values reaching 3.39, 1.81, and 1.34 times the control value, respectively (Fig. 5). These results suggest that proline accumulation was significantly induced by salt, drought, and ABA stress treatments in lily and that the rapid increase in *LhSorP5CS* expression may be involved in proline production as part of lily’s stress response.

## Discussion

Proline, an amino acid known to function as an osmoprotectant, a ROS scavenger, a cell structure maintainer, and a signaling molecule, plays an active role in plant stress responses. In plants, the rate-limiting steps in proline synthesis are catalyzed by P5CS. The function of the first plant *P5CS* gene from *Vigna aconitifolia* was revealed by its complementation of the *proA* and *proB* mutations in *E. coli* (Hu et al. 1992). Since then, *P5CS* homologues have been cloned from *A. thaliana* (Strizhov et al. 1997), *O. sativa* (Igarashi et al. 1997), *M. truncatula* (Ginzberg et al. 1998) and other species (Chen et al. 2010; Fujita et al. 1998; Zhu et al. 2012). In this study, we cloned the full-length cDNA sequence of the *LhSorP5CS* gene, the first *P5CS* gene reported in *Lilium*. Phylogenetic analysis revealed that LhSorP5CS was closely related to P5CS proteins from monocot plants, and had 83% identity with MaAAAP5CS. The *LhSorP5CS* transcript level was significantly up-regulated by salt, drought, and ABA stress. By 12 h after stress treatments, a significantly higher proline content was detected in leaf tissue, revealing proline accumulation induced by salt and drought stress in lily. This agrees with previous research that showed the accumulation of *P5CS* transcripts and proline in many different plant species under stress. In sweet sorghum (*Sorghum bicolor*) (Su et al. 2011), induction of *P5CS* expression was observed before proline accumulation under salt and drought stress. Similarly, rapid up-regulation of *PvP5CS* was observed prior to proline accumulation in *Phaseolus vulgaris* (Chen et al. 2009). Hence, it appears that *LhSorP5CS* is involved in proline synthesis and that the accumulation of proline might result from up-regulation of *LhSorP5CS* expression in lily.

Because *P5CS* genes appear to be the most important genes involved in catalyzing proline synthesis, they have been selected as candidate genes to improve abiotic stress tolerance by elevating in vivo proline levels. Increased proline production and enhanced tolerance to multiple stresses have been reported in several transgenic plants, including potato (*Solanum tuberosum*) (Hmida-Sayari et al. 2005), Arabidopsis (Chen et al. 2013), chickpea (*Cicer arietinum*) (Ghanti et al. 2011), and wheat (*Triticum aestivum*) (Vendruscolo et al. 2007). The *LhSorP5CS* gene that we cloned in the present study is therefore also a candidate gene for future transgenic research in lily.

In higher plants, stress-induced proline accumulation has been observed in many species, and halophytes generally accumulated more proline than glycophytes under adverse conditions (Verbruggen and Hermans 2008). Therefore, the proline level was often correlated with stress tolerance, especially for salt and drought tolerance (Chakraborty et al. 2012; Chauhan et al. 1980; Putnik-Delic et al. 2013). However, this correlation was not always consistent. A recent study of rice seedlings suggested that the proline accumulation level was not a reliable parameter for assessing tolerance of saline alkaline conditions (Lv et al. 2015). Similarly, a study by Deng (2013) suggested that there was no correlation between proline accumulation and drought tolerance in ten genotypes of Tibetan hull-less barley (*Hordeum vulgare* var. *nudum*). Hence, it is worth noting that even though proline plays a multifaceted role in plant stress adaption, its accumulation level may not be significantly associated with stress tolerance and its role may differ among species. It will be necessary to confirm this association in future research on other lily accessions.

The non-consistent correlation between proline level and stress tolerance can be ascribed to the fact that proline turnover, rather that just proline accumulation, was more important to plant growth maintaining under stress. Although many early attempts have proven that improvement of stress tolerance through elevating proline level was feasible, recent evidence (Bhaskara 2015) showed that mutation of *proline dehydrogenase 1* (*PDH1*), the key enzyme catalyze proline catabolism, resulted in similar reduction in growth as the *p5cs1* mutant in spite of elevated proline production. Such findings emphasize the importance of proline catabolism to plant growth maintaining under stress, and underlie that the flux and turnover of proline, rather than just proline accumulation, might be more important to stress adaption for plants.

## Conclusions

*LhSorP5CS*, the first *P5CS* gene cloned from *Lilium*, encoded a protein with 712 amino acids and a pI of 5.71. Expression of *LhSorP5CS* was significantly up-regulated when the plants were exposed to salt, drought, and ABA stresses. Proline accumulation increased significantly 12 h after the stress treatments. These findings suggest that *LhSorP5CS* plays an important role in proline synthesis and stress tolerance in lily. However, there should be other *P5CS* homologues in the lily genome, and some of them could be highly stress-induced and more important for proline accumulation. Future studies on *P5CS* family genes would be of great importance to proline-related stress tolerance in lily.

## Additional files

**Additional file 1: Figure S1.** Multiple sequence alignment of LhSorP5CS with P5CSs from other plants. Alignment of the putative amino acid sequence of the Oriental hybrid lily cv. Sorbonne LhSorP5CS with sequences of *Arabidopsis thaliana* AtP5CS1 (Genbank accession no. NP181510) and AtP5CS2 (Genbank accession no. NP191120), and *Oryza sativa* OsP5CS1 (Genbank accession no. BAA19916) and OsP5CS2 (Genbank accession no. NP001044802). Identical residues are shaded in dark blue, highly similar residues are shaded in pink and similar residues are shaded in light blue. Upperlined sequences represent putative ATP and NAD(P)H-binding sites, conserved Glu-5-kinase and GSA-DH domains, and leucine (Leu)-rich regions. The conserved phenylalanine (Phe) residue that functions in proline feedback inhibition is indicated by an asterisk (*).

**Additional file 2: Figure S2.** Ramachandran plot of the built model of LhSorP5CS. The red, yellow, light yellow and white regions represent the most favored, additional allowed, generously allowed, and disallowed regions respectively.

## Abbreviations

P5CS: Δ1-pyrroline-5-carboxylate synthetases
GSA: γ-glutamate-semialdehyde
RACE: rapid amplification of cDNA ends
ABA: abscisic acid
ORF: open reading frame
UTR: untranslated region

## References

[CR1] Adams E, Frank L (1980). Metabolism of proline and the hydroxyprolines. Annu Rev Biochem.

[CR2] Bates LS, Waldren RP, Teare ID (1973). Rapid determination of free proline for water-stress studies. Plant Soil.

[CR3] Bhaskara GB, Yang TH, Verslues PE (2015) Dynamic proline metabolism: importance and regulation in water limited environments. Front Plant Sci 6:484 10.3389/fpls.2015.00484 PMC4479789 26161086

[CR4] Chakraborty K, Sairam RK, Bhattacharya RC (2012). Salinity-induced expression of pyrrolline-5-carboxylate synthetase determine salinity tolerance in Brassica spp. Acta Physiol Plant.

[CR5] Chauhan RPS, Chauhan CPS, Kumar D (1980). Free proline accumulation in cereals in relation to salt tolerance. Plant Soil.

[CR6] Chen JB, Wang SM, Jing RL, Mao XG (2009). Cloning the PvP5CS gene from common bean (*Phaseolus vulgaris*) and its expression patterns under abiotic stresses. J Plant Physiol.

[CR7] Chen JB, Zhang XY, Jing RL, Blair MW, Mao XG, Wang SM (2010). Cloning and genetic diversity analysis of a new P5CS gene from common bean (*Phaseolus vulgaris* L.). Theor Appl Genet.

[CR8] Chen JB, Yang JW, Zhang ZY, Feng XF, Wang SM (2013). Two P5CS genes from common bean exhibiting different tolerance to salt stress in transgenic *Arabidopsis*. J Genet.

[CR9] Chinnusamy V, Jagendorf A, Zhu JK (2005). Understanding and improving salt tolerance in plants. Crop Sci.

[CR10] Delauney AJ, Verma DPS (1993). Proline biosynthesis and osmoregulation in plants. Plant J.

[CR11] Deng G, Liang J, Xu D, Long H, Pan Z, Yu M (2013). The relationship between proline content, the expression level of P5CS (delta(1)-pyrroline-5-carboxylate synthetase), and drought tolerance in Tibetan hulless barley (*Hordeum vulgare* var. nudum). Russ J Plant Physiol.

[CR12] Fabro G, Kovacs I, Pavet V, Szabados L, Alvarez ME (2004). Proline accumulation and AtP5CS2 gene activation are induced by plant-pathogen incompatible interactions in *Arabidopsis*. Mol Plant Microbe Interact.

[CR13] Fourie JJ, Joubert A, Labuschagne M, Beugnet F (2014). New method using quantitative PCR to follow the tick blood meal and to assess the anti-feeding effect of topical acaricide against *Rhipicephalus sanguineus* on dogs. Comp Immunol Microbiol Infect Dis.

[CR14] Fujita T, Maggio A, Garcia-Rios M, Bressan RA, Csonka LN (1998). Comparative analysis of the regulation of expression and structures of two evolutionarily divergent genes for delta(1)-pyrroline-5-carboxylate synthetase from tomato. Plant Physiol.

[CR15] Ghanti SKK, Sujata KG, Kumar BMV, Karba NN, Reddy KJ, Rao MS, Kishor PBK (2011). Heterologous expression of P5CS gene in chickpea enhances salt tolerance without affecting yield. Biol Plant.

[CR16] Ginzberg I, Stein H, Kapulnik Y, Szabados L, Strizhov N, Schell J, Koncz C, Zilberstein A (1998). Isolation and characterization of two different cDNAs of delta(1)-pyrroline-5-carboxylate synthase in alfalfa, transcriptionally induced upon salt stress. Plant Mol Biol.

[CR17] Hmida-Sayari A, Gargouri-Bouzid R, Bidani A, Jaoua L, Savoure A, Jaoua S (2005). Overexpression of delta(1)-pyrroline-5-carboxylate synthetase increases proline production and confers salt tolerance in transgenic potato plants. Plant Sci.

[CR18] Hu CAA, Delauney AJ, Verma DPS (1992). A bifunctional enzyme (delta-1-pyrroline-5-carboxylate synthetase) catalyzes the 1st 2 steps in proline biosynthesis in plants. Proc Natl Acad Sci USA.

[CR19] Igarashi Y, Yoshiba Y, Sanada Y, YamaguchiShinozaki K, Wada K, Shinozaki K (1997). Characterization of the gene for delta(1)-pyrroline-5-carboxylate synthetase and correlation between the expression of the gene and salt tolerance in *Oryza sativa* L. Plant Mol Biol.

[CR20] Krissinel E (2007). On the relationship between sequence and structure similarities in proteomics. Bioinformatics.

[CR21] Kumar V, Shriram V, Kishor PBK, Jawali N, Shitole MG (2010). Enhanced proline accumulation and salt stress tolerance of transgenic indica rice by over-expressing P5CSF129A gene. Plant Biotechnol Rep.

[CR22] Laskowski RA, Macarthur MW, Moss DS, Thornton JM (1993). Procheck—a program to check the stereochemical quality of protein structures. J Appl Crystallogr.

[CR23] Liang XW, Zhang L, Natarajan SK, Becker DF (2013). Proline mechanisms of stress survival. Antioxid Redox Signal.

[CR24] Lv BS, Ma HY, Li XW, Wei LX, Lv HY, Yang HY, Jiang CJ, Liang ZW (2015). Proline accumulation is not correlated with saline–alkaline stress tolerance in rice seedlings. Agron J.

[CR25] Maggio A, Miyazaki S, Veronese P, Fujita T, Ibeas JI, Damsz B, Narasimhan ML, Hasegawa PM, Joly RJ, Bressan RA (2002). Does proline accumulation play an active role in stress-induced growth reduction?. Plant J.

[CR26] Matysik J, Alia B Bhalu, Mohanty P (2002). Molecular mechanisms of quenching of reactive oxygen species by proline under stress in plants. Curr Sci India.

[CR27] Molinari HBC, Marur CJ, Bespalhok JC, Kobayashi AK, Pileggi M, Leite RP, Pereira LFP, Vieira LGE (2004). Osmotic adjustment in transgenic citrus rootstock Carrizo citrange (*Citrus sinensis* Osb. × *Poncirus trifoliata* L. Raf.) overproducing proline. Plant Sci.

[CR28] Putnik-Delic M, Maksimovic I, Venezia A, Nagl N (2013). Free proline accumulation in young sugar beet plants and in tissue culture explants under water deficiency as tools for assessment of drought tolerance. Rom Agric Res.

[CR29] Rai AN, Penna S (2013). Molecular evolution of plant P5CS gene involved in proline biosynthesis. Mol Biol Rep.

[CR30] Schwede T, Kopp J, Guex N, Peitsch MC (2003). SWISS-MODEL: an automated protein homology-modeling server. Nucleic Acids Res.

[CR31] Sharma S, Villamor JG, Verslues PE (2011). Essential role of tissue-specific proline synthesis and catabolism in growth and redox balance at low water potential. Plant Physiol.

[CR32] Shinde S, Villamor JG, Lin W, Sharma S, Verslues PE (2016). Proline coordination with fatty acid synthesis and redox metabolism of chloroplast and mitochondria. Plant Physiol.

[CR33] Strizhov N, Abraham E, Okresz L, Blickling S, Zilberstein A, Schell J, Koncz C, Szabados L (1997). Differential expression of two P5CS genes controlling proline accumulation during salt-stress requires ABA and is regulated by ABA1, ABI1 and AXR2 in *Arabidopsis*. Plant J.

[CR34] Su M, Li XF, Ma XY, Peng XJ, Zhao AG, Cheng LQ, Chen SY, Liu GS (2011). Cloning two P5CS genes from bioenergy sorghum and their expression profiles under abiotic stresses and MeJA treatment. Plant Sci.

[CR35] Szabados L, Savoure A (2010). Proline: a multifunctional amino acid. Trends Plant Sci.

[CR36] Tamura K, Dudley J, Nei M, Kumar S (2007). MEGA4: molecular evolutionary genetics analysis (MEGA) software version 4.0. Mol Biol Evol.

[CR37] Tan CM, Chen RJ, Zhang JH, Gao XL, Li LH, Wang PR, Deng XJ, Xu ZJ (2013). OsPOP5, a prolyl oligopeptidase family gene from rice confers abiotic stress tolerance in *Escherichia coli*. Int J Mol Sci.

[CR38] Trovato M, Mattioli R, Costantino P (2008). Multiple roles of proline in plant stress tolerance and development. Rend Lincei.

[CR39] Vendruscolo ECG, Schuster I, Pileggi M, Scapim CA, Correa Molinari HB, Marur CJ, Esteves Vieira LG (2007). Stress-induced synthesis of proline confers tolerance to water deficit in transgenic wheat. J Plant Physiol.

[CR40] Verbruggen N, Hermans C (2008). Proline accumulation in plants: a review. Amino Acids.

[CR41] Verslues PE, Agarwal M, Katiyar-Agarwal S, Zhu J, Zhu JK (2006). Methods and concepts in quantifying resistance to drought, salt and freezing, abiotic stresses that affect plant water status. Plant J.

[CR42] Yamagishi M (2011). Oriental hybrid lily Sorbonne homologue of LhMYB12 regulates anthocyanin biosyntheses in flower tepals and tepal spots. Mol Breed.

[CR43] Zhu JK (2001). Plant salt tolerance. Trends Plant Sci.

[CR44] Zhu XY, Li XP, Zou Y, Chen WX, Lu WJ (2012). Cloning, characterization and expression analysis of delta(1)-pyrroline-5-carboxylate synthetase (P5CS) gene in harvested papaya (*Carica papaya*) fruit under temperature stress. Food Res Int.

