# Supplementary figures and images for: Characterization of LhSorP5CS, a gene catalyzing proline synthesis in Oriental hybrid lily Sorbonne: molecular modelling and expression analysis
Source: Bot Stud. 2017 Jan 18;58:10. doi: 10.1186/s40529-017-0163-0 (PMC5432930; doi:10.1186/s40529-017-0163-0)

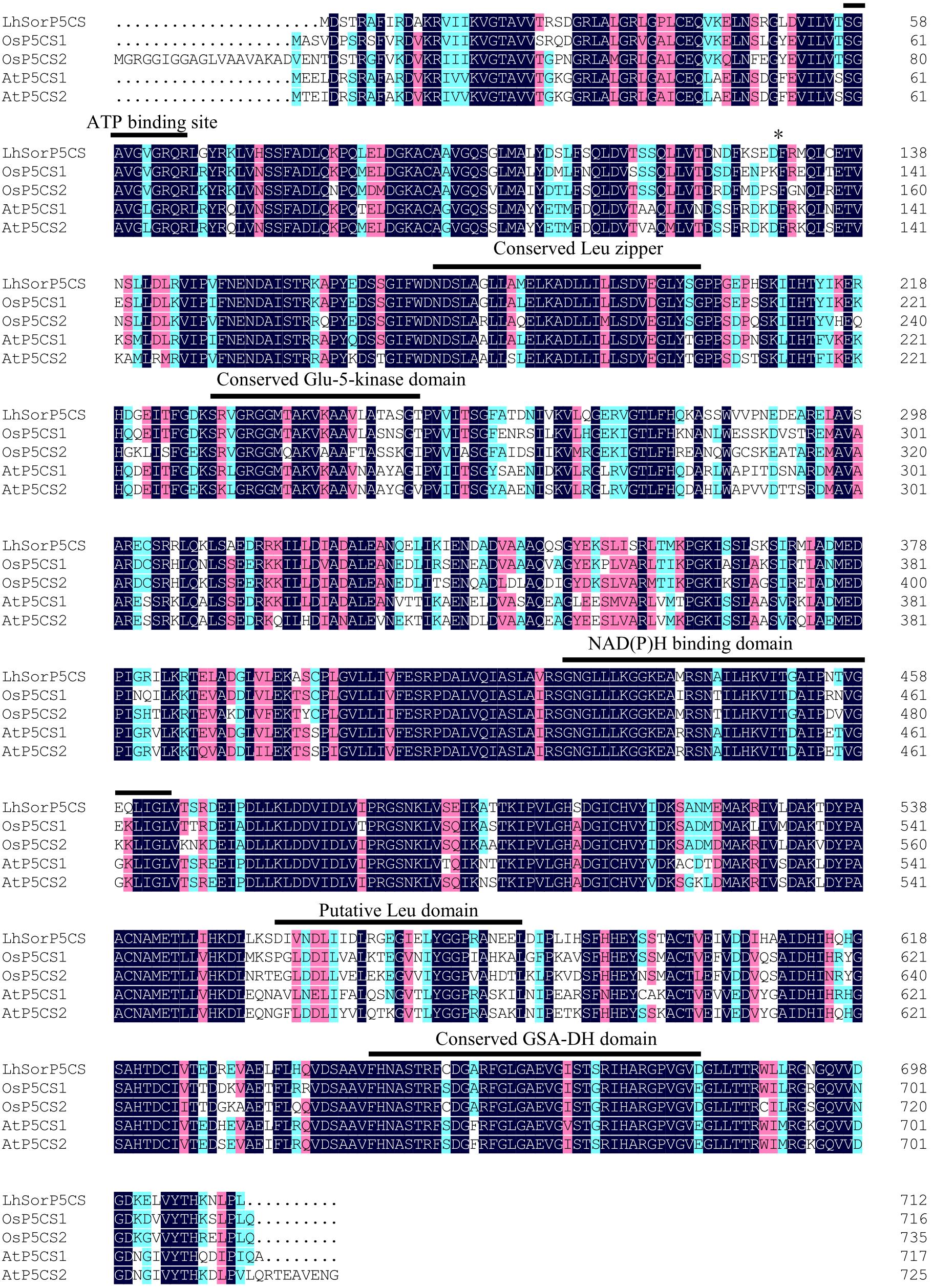

Supplement: Supplementary file 1 — Additional file 1: Figure S1. Multiple sequence alignment of LhSorP5CS with P5CSs from other plants. Alignment of the putative amino acid sequence of the Oriental hybrid lily cv. Sorbonne LhSorP5CS with sequences of Arabidopsis thaliana AtP5CS1 (Genbank accession no. NP181510) and AtP5CS2 (Genbank accession no. NP191120), and Oryza sativa OsP5CS1 (Genbank accession no. BAA19916) and OsP5CS2 (Genbank accession no. NP001044802). Identical residues are shaded in dark blue, highly similar residues are shaded in pink and similar residues are shaded in light blue. Upperlined sequences represent putative ATP and NAD(P)H-binding sites, conserved Glu-5-kinase and GSA-DH domains, and leucine (Leu)-rich regions. The conserved phenylalanine (Phe) residue that functions in proline feedback inhibition is indicated by an asterisk (*). [file 40529_2017_163_MOESM1_ESM.jpg]

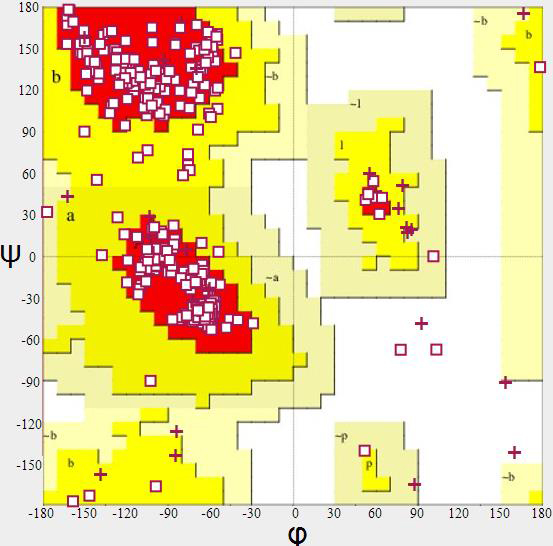

Supplement: Supplementary file 2 — Additional file 2: Figure S2. Ramachandran plot of the built model of LhSorP5CS. The red, yellow, light yellow and white regions represent the most favored, additional allowed, generously allowed, and disallowed regions respectively. [file 40529_2017_163_MOESM2_ESM.jpg]
